# Patient preferences for development in MRI scanner design: a survey of claustrophobic patients in a randomized study

**DOI:** 10.1007/s00330-020-07060-9

**Published:** 2020-09-02

**Authors:** Elisa Iwan, Jinhua Yang, Judith Enders, Adriane Elisabeth Napp, Matthias Rief, Marc Dewey

**Affiliations:** 1 Departement of Anaesthesiology, Charité Berlin, Charitéplatz 1, 10117 Berlin, Germany; 2 Department of Radiology, Charité Berlin, Charitéplatz 1, 10117 Berlin, Germany; 3 Pengzhou, China; 4 Department of Radiology, Campus Virchow-Klinikum Klinik für Pädiatrie m.S. Onkologie und Hämatologie, Charité Berlin, Mittelallee 8, 4. OG Augustenburger Platz 1, 13353 Berlin, Germany

**Keywords:** Claustrophobia, Magnetic resonance imaging, Questionnaire

## Abstract

**Objective:**

To investigate which magnetic resonance imaging (MRI) scanner designs claustrophobic patients prefer.

**Material/methods:**

We analyzed questionnaires completed by 160 patients at high risk for claustrophobia directly after a scan in either a short-bore or open panoramic scanner as part of a prospective randomized trial Enders et al (BMC Med Imaging 11:4, 2011). Scanner preferences were judged based on schematic drawings of four scanners. Information on the diagnostic performance of the depicted scanners was provided, too.

**Results:**

A majority of patients suggested upright open (59/160, 36.9%) and open panoramic (53/160, 33.1%) before short-bore designs (26/160, 16.3%, for all *p* < 0.001) for future development. When asked about patients’ preferred scanner choice for an upcoming examination, information about a better diagnostic performance of a short-bore scanner significantly improved its preference rates (from 6/160 to 49/160 or 3.8 to 30.5%, *p* < 0.001). Patients with a claustrophobic event preferred open designs significantly more often than patients without a claustrophobic event (*p* = 0.047). Patients scanned in a short-bore scanner in our trial preferred this design significantly more often (*p* = 0.003). Noise reduction (51/160, 31.9%), more space over the head (44/160, 27.5%), and overall more space (33/160, 20.6%) were the commonest suggested areas of improvement.

**Conclusion:**

Patients at high risk for claustrophobia visually prefer open- over short-bore MRI designs for further development. Education about a better diagnostic performance of a visually less-attractive scanner can increase its acceptance. Noise and space were of most concern for claustrophobic patients. This information can guide individual referral of claustrophobic patients to scanners and future scanner development.

**Key Points:**

*• Patients at high risk for claustrophobia visually favor the further development of open scanners as opposed to short- and closed-bore scanner designs.*

*• Educating claustrophobic patients about a higher diagnostic performance of a short-bore scanner can significantly increase their acceptance of this otherwise visually less-attractive design.*

*• A medical history of earlier claustrophobic events in a given MRI scanner type and focusing on the features “more space” and “noise reduction” can help to guide referral of patients who are at high risk for claustrophobia.*

**Electronic supplementary material:**

The online version of this article (10.1007/s00330-020-07060-9) contains supplementary material, which is available to authorized users.

## Introduction

Claustrophobia is a common problem in magnetic resonance imaging (MRI) and has been defined as the combined fear of suffocation and restriction [2]. It is estimated to occur in 2.1 to 14.3% of all MRI examinations [3–6]. Negative consequences for patients range from the need for conscious sedation to the avoidance of important MRI screening examinations that might offer patients the chance of early diagnosis and treatment of certain diseases [7–10].

Claustrophobia is influenced by many factors such as sex, positioning in the scanner, body weight, and the shape of the scanner [3, 5, 10, 12]. Many techniques such as the introduction of silent gradients, additional light in the bore, special glasses, and virtual reality tools [13] have been successfully introduced into clinical routine to relieve claustrophobia. Older generation scanners featuring closed, rather narrow, and long bores can trigger a claustrophobic experience [11, 14]. One approach to reduce claustrophobia has been to design scanners with a less-restrictive architecture [11, 14–16].

The first visual impression of an MRI scanner is a relevant variable associated with the occurrence of claustrophobia. Claustrophobic reactions are related to cognitions such as “harm caused by the machine”; moreover, a considerable number of claustrophobic events, including premature termination of the MRI examination, occur upon merely looking at the scanner before the actual examination and experience of lying within the scanner have even started [17–19]. Therefore, it has been suggested that patient comfort should drive the development of new scanners [20].

To this end, new scanners, e.g., with wider and even shorter bores or with an open design that provide a lateral panoramic view, have been developed and are increasingly used these days. The use of such scanners in clinical routine contributed to the reduction of claustrophobic events and increased patient comfort in past studies [5, 21, 22].

So far, however, in patients at high risk for experiencing claustrophobia, neither an open- nor a short-bore scanner could decrease the claustrophobic event rate significantly [19]. Moreover, the new scanners mentioned above are rather expensive and not necessarily widely available. With this in mind, it is worthwhile to investigate scanner preferences of patients at high risk for claustrophobia and search for factors that might influence these preferences, such as the visual perception of and associated cognitions with a given MRI scanner design.

The aim of this study is therefore to investigate which MRI scanner features patients who are at a higher risk for claustrophobia prefer. This can guide further industrial scanner development and the clinical use of MRI scanners with features likely to reduce claustrophobia in patients at risk.

## Material and methods

### Ethics statement

Approval was obtained from the Institutional Review Board of Charité, Berlin. All patients were educated about the conduct and purpose of the study and gave written informed consent prior to randomization.

### Study design and conduct

**Fig. 1:**
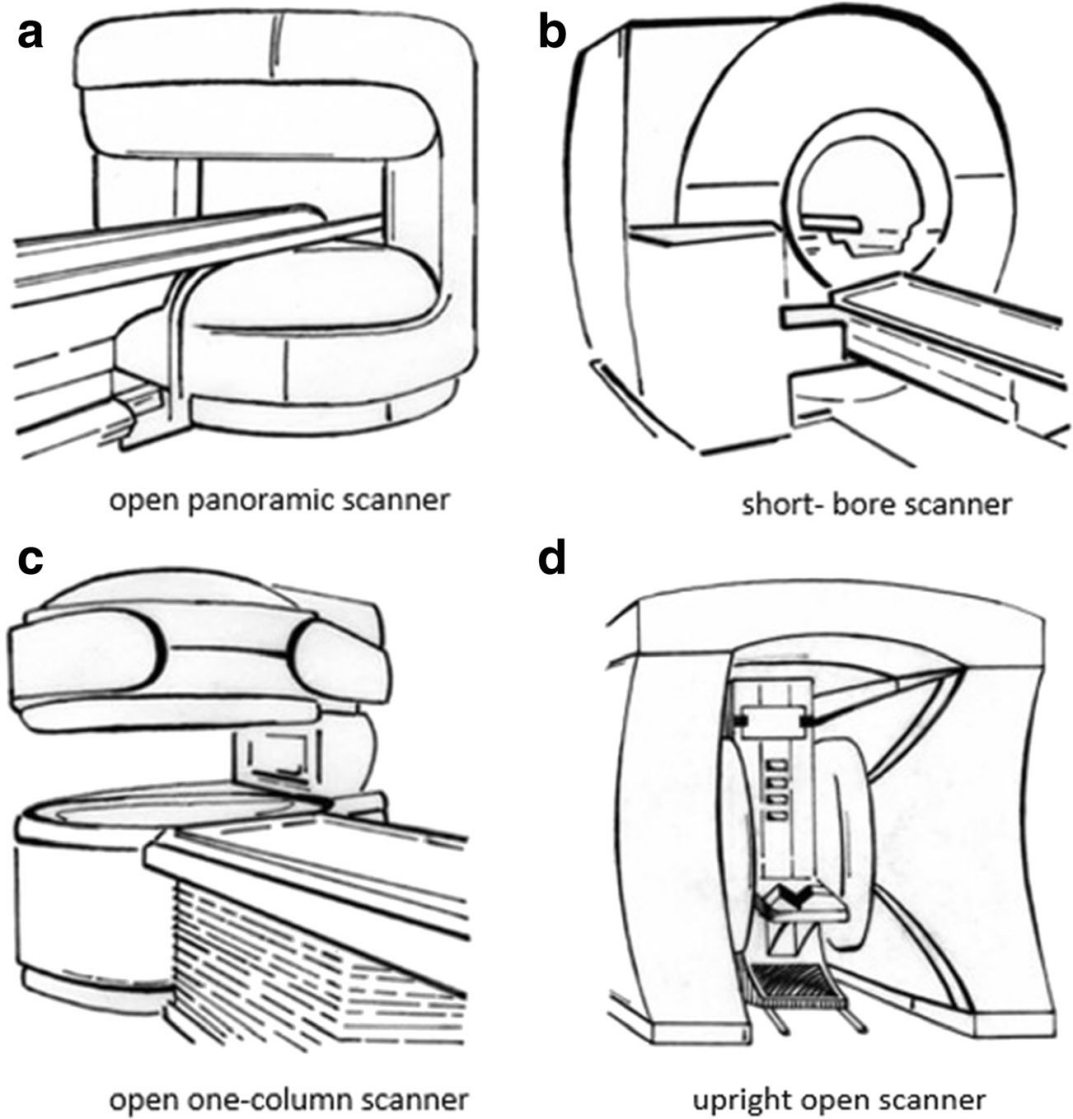
MRI scanner designs in the study questionnaire to choose from. Four different MRI scanner designs were presented to choose from when asked about their design preferences in the questionnaire. **a** An open panoramic scanner with a vertical magnetic field and 1-T field strength (Phillips, Panorama). **b** A short-bore scanner with 1.5-T field strength (Siemens, MAGNETOM Avanto). **c** A 0.4-T open one-column scanner. **d** A 0.6-T frontal and overhead open scanner in which the patient sits in an upright position. The following additional information on the diagnostic utility of the different scanner designs was given in a second step: (**a**) good, (**b**) very good, (**c**) moderate, and (**d**) adequate diagnostic utility

**Fig. 2:**
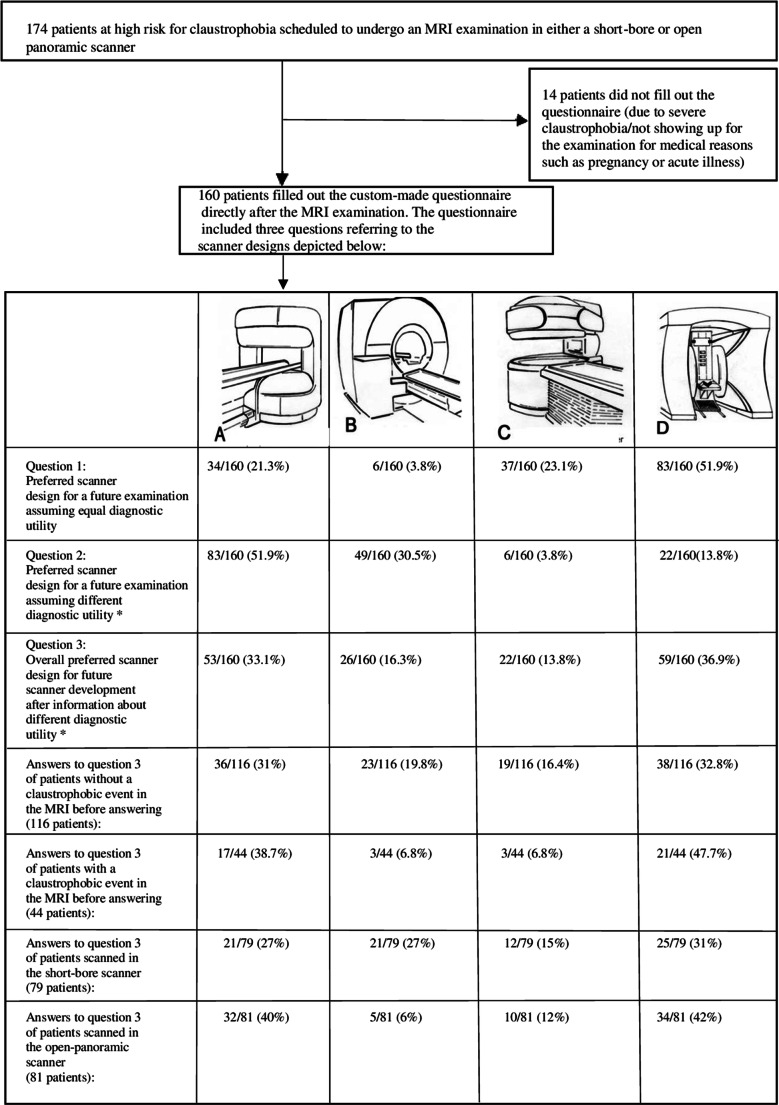
Conduct of the trial. A total of 174 patients were initially included. Fourteen patients did not fill out the questionnaire due to severe claustrophobia or because they did not undergo the MRI examination due to medical reasons. A total of 160 patients answered questions 1–4 regarding design preferences after the MRI examination. Question 1 and 2 asked about the preferred scanner design for an imaginary future examination as judged by the drawings in the questionnaire assuming equal (question 1) versus different diagnostic utility* (question 2) of the depicted scanners. Question 3 queried about the overall preferred scanner design for future development by manufacturers. Differences in answers to question 3 depending on the occurrence of a claustrophobic event and the scanner type used in the examination preceding the questionnaire are also shown. Question 4 was an open question in which patients could make suggestions for further improvements of MRI scanners. Answers to question 4 are not shown in this presentation (for details, see Fig. 6). *Differences in diagnostic utility: **a** good, **b** very good, **c** moderate, and **d** adequate

To investigate which scanner design patients who are at high risk for claustrophobia prefer, we analyzed feedback questionnaires filled out by patients enrolled in the prospective randomized controlled “CLAUSTRO” trial [1]. The trial included 174 patients at high risk for claustrophobia as judged by past experiences and claustrophobia-related scores such as the Claustrophobia Questionnaire (CLQ) score and took place between June 19, 2008, and August 14, 2009 (for details, see [1, 19]). All patients included had a clinical indication for MRI. They were randomly assigned to undergo an examination in either a new open panoramic MRI scanner (Panorama, Philips Medical Systems) or a short-bore scanner (Siemens, Magnetom Avanto, Siemens Medical Solutions). Previous patient experiences concerning scanner design were not considered in the allocation process. Baseline characteristics of the two groups were matched. Silent sequences were not used in our study and headphones and/or ear plugs were given to patients (when technically feasible) upon request. Directly after the examination, they were supposed to fill out a custom-made questionnaire that addressed their preferences for future scanner design (Fig. 1; Supplementary Appendix). A claustrophobic event was defined as the inability to complete imaging in the assigned scanner. For details of the conduct of the randomized controlled trial and study protocol, see Enders et al [19]. Of the 160 patients who completed the questionnaire, 44 (27.5%) experienced a claustrophobic event in the preceding examination (Fig. 2).

### Scanner design questionnaire

Patients filled out the custom-made questionnaire directly after their MRI examination. The data were then consolidated in an electronic database together with other patient data such as the occurrence of a claustrophobic event. The scanner design questionnaire included four drawings of different MRI scanner designs and four related questions (Fig. 1; Supplementary Appendix). The first two questions were about patient preferences for a possible future MRI examination. First, patients were asked about preferences assuming equal diagnostic performance of the depicted scanners. Thereafter, patients were again asked about their preferences after having been informed about differences in diagnostic performance of the scanners. Ranking of diagnostic performance from best to worst was as follows: short-bore scanner, open panoramic scanner, one-column design, and upright open scanner. Thereafter, patients could indicate which of the schematically depicted scanner designs they wished to be further developed in terms of diagnostic performance and/or design by manufacturers.

In an additional open question, patients could suggest general improvements for MRI scanners to find out which features they subjectively regarded as most important to relieve claustrophobia.

### Outcomes measures

Our outcome measures were which scanner features patients at high risk for claustrophobia visually preferred and which factors (such as education about diagnostic performance or former scanner experiences) contributed to their preference. We chose the second outcome measure because cognitions and attitudes towards an MRI scanner even before the actual experience of lying within the magnet were shown to contribute to feelings of claustrophobia [17–19]. Knowledge about MRI preferences of a population of patients at high risk for claustrophobia might be valuable for further scanner development as well as for the individual assignment of high-risk patients to new patient-centered scanners or scanners with specific features.

### Statistical analysis

Statistical analysis was done using R (Version 3.4.1) and IBM SPSS Statistics 23. A multinominal chi-square test of goodness fits was performed as appropriate for categorical variables. For intraindividual comparison of the influence of the information about the diagnostic utility of the scanners on preferences, the test for marginal homogeneities was used. A 95% multinominal proportion confidence interval was chosen. Pearson’s chi-square test was used to analyze the influence of the presence of a claustrophobic event and other scanner type patients were scanned in had on design preferences.

## Results

Of the 174 enrolled patients, 160 (92%) completed the custom-made questionnaire. Fourteen patients (8%) did not fill out the questionnaire due to medical contraindications to taking part in the trial such as pregnancy or acute illness or due to severe claustrophobia after the examination.

### Relationship between preference and information on diagnostic performance

When assuming that all scanners had the same diagnostic performance and that patients could pick a scanner design for an imaginary upcoming MRI examination in the near future, 51.9% of patients (83/160) favored an upright open scanner over an open one-column design (23.1%; 37/160) and an open panoramic scanner design (21.3%; 34/160). Only 3.8% (6/160) of patients chose the short-bore scanner in this scenario.

After having been educated about a better diagnostic performance of the short-bore and open panoramic scanners, patients significantly changed their design preferences. In this scenario, 51.9% (83/160) of patients preferred the open panoramic scanner followed by the short-bore scanner (30.5%; 49/160). The upright open and open one-column scanners were only chosen by 13.8% and 3.8%, respectively.

**Fig. 3:**
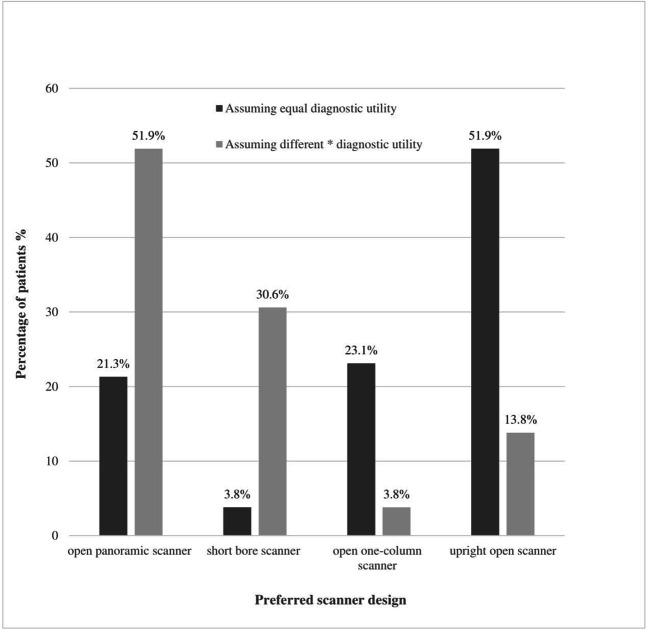
Scanner design preferences in relation to the diagnostic performance of a scanner. When patients did not have any information on the different diagnostic utilities of the scanners depicted in the questionnaire, and therefore assumed equal diagnostic utility of all scanner designs to choose from, a majority of patients preferred an upright open scanner (51.9%). The short-bore scanner was least frequently chosen in this scenario (3.8%). After having been informed about the superior diagnostic performance of the short-bore and open panoramic scanner compared with the other imagers, patients favored those scanners significantly more often (51.9% for the open panoramic scanner and 30.6% for the short-bore scanner, respectively, *p* < 0.001) than before information about the diagnostic utility of the individual scanners was provided. *The difference in diagnostic utility was defined as follows: open panoramic scanner: good; short-bore scanner: very good; open one-column scanner: moderate; upright open scanner: adequate

The changes in scanner preferences following information about different diagnostic performances of the scanners were statistically significant (for all *p* < 0.001; Table 1; Fig. 3): for the open panoramic scanner (21.2 to 51.9%), short-bore scanner (3.8 to 30.5%), open one-column scanner (23.1 to 13.8%), and upright open scanner (51.9 to 13.8%).

**Table 1:** Influence of the diagnostic performance of a scanner on design preferences

Preferred scanner design for a possible future examination	Assuming equal diagnostic utility		Assuming different diagnostic utilities		Change in preferences after education about different diagnostic utilities
					*p* < 0.001**
	*n* = 160	95% CI	*n* = 160	95% CI	
A: Open panoramic scanner, *n* (%)	34 (21.2)	15.6–28.2	83 (51.9)	44.2–59.9	
B: Short-bore scanner, *n* (%)	6 (3.8)	1.7–7.9	49 (30.5)	24.0–38.2	
C: Open one-column scanner, *n* (%)	37 (23.1)	17.3–30.2	6 (3.8)	1.7–7.9	
D: Upright open scanner, *n* (%)	83 (51.9)	44.2–59.5	22 (13.8)	9.3–19.9	

Responses of patients concerning MRI scanner designs in relation to information about the diagnostic utility of the scanners to choose from directly after an examination in either a short-bore or open panoramic scanner

Question 1: Preferred scanner design for a future MRI examination when assuming equal diagnostic utility

Question 2: Preferred scanner design for a future MRI examination when assuming different diagnostic utilities: A = “good diagnostic utility,” B = “very good [...],” C = “moderate [...],” D = “adequate” [..]

The change in difference in design preferences in relation to information about a different diagnostic utility of the scanners was statistically significant (*p* < 0.001)

**p value calculated with test for marginal homogeneities

### Preferred scanner design for further development

This question was asked after taking all information into account (including differences in diagnostic performance) and aimed at preferences for future scanner development as opposed to preferences for scanner designs for an individual upcoming MRI exam. 36.9% of patients (*n*/total = 59/160) preferred an upright open scanner and 33.1% (53/160) an open panoramic scanner design. Further development of the short-bore scanner was less frequently suggested (16.3%; 26/160). Only 13.8% of patients (22/160) favored the open scanner with a one-column design (Table 2; Fig. 2.)

**Table 2:** Preferred scanner design for further development by manufacturers

Preferred scanner design for future development			Distribution of proportions in answers
			*p* = <0.001*
	*n*/160 patients	95% CI	
A: Open panoramic scanner, *n* (%)	53/160 (33.1)	25.6–41.3	
B: Short-bore scanner, *n* (%)	26/160 (16.3)	8.8–25	
C: Open one-column scanner, *n* (%)	22/160 (13.8)	6.3–22.5	
D: Upright open scanner, *n* (%)	59/ 160 (36.9)	29.4–45.6	

When asked about their preferred scanner design for further development by manufacturers, a majority of patients suggested an upright open or open one-column scanner design

*A four-sample test of equality of proportions showed a non-random distribution of answers (*p* < 0.001)

### Influence of the occurrence of a claustrophobic event on design preferences

**Fig. 4:**
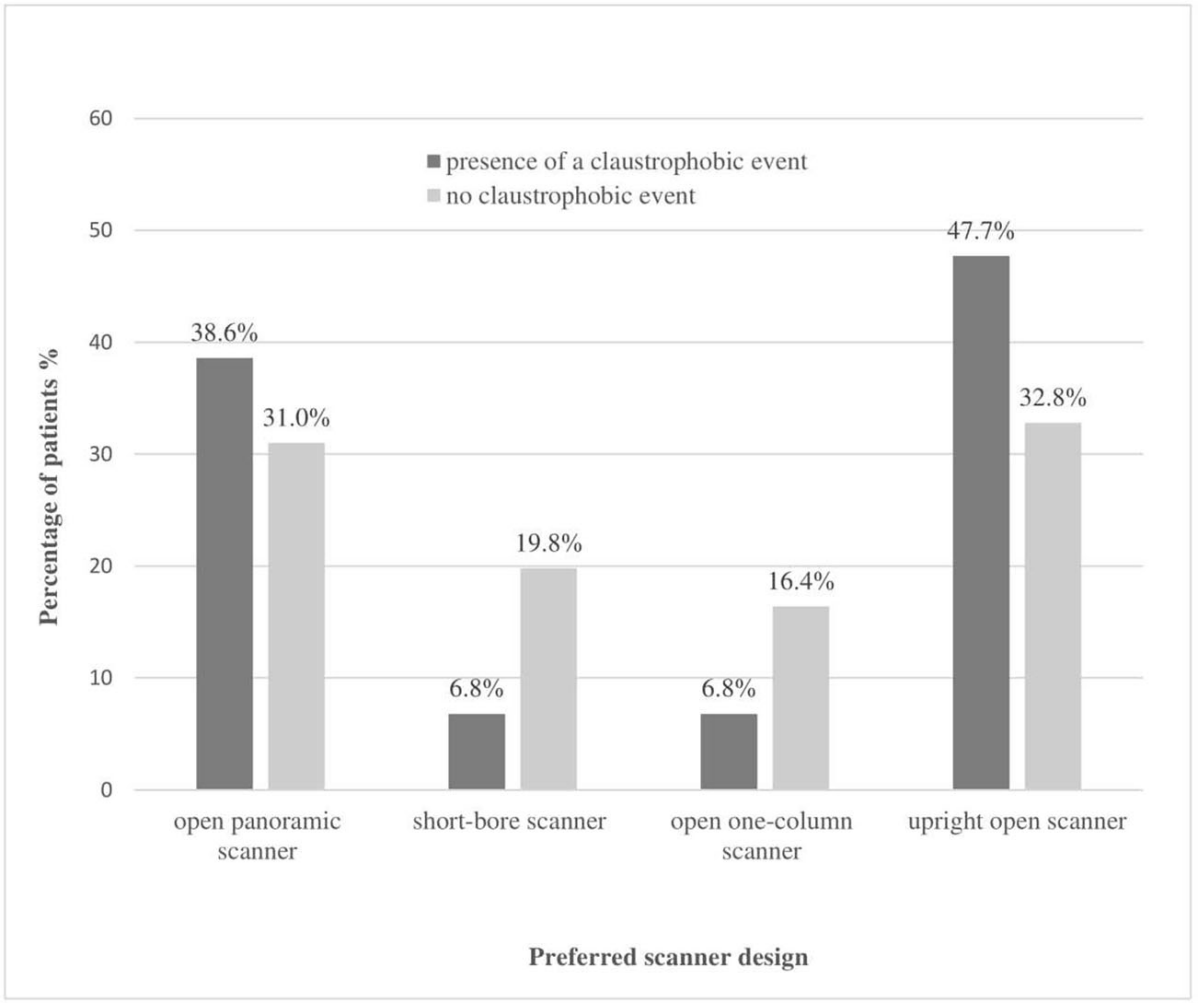
Scanner preference in relation to occurrence of a claustrophobic event in the preceding MRI examination. Patients who experienced a claustrophobic event immediately before filling out the questionnaire preferred an upright open or open panoramic design significantly more often than patients without a claustrophobic event (*p* = 0.047). Only 6.8% of patients with an event favored a short-bore scanner design. Patients without a claustrophobic event were more likely to accept a short-bore scanner design (19.8%)

The occurrence of a claustrophobic event in the preceding MRI examination had a significant influence on design preferences for future scanner development. 86.4% of patients who had a claustrophobic event immediately before filling out the questionnaire preferred an upright open scanner (47.7%; 21/44) or an open panoramic scanner (38.7%; 17/44). Only 6.8% (3/44) favored a short-bore or open one-column scanner (6.8%; 3/44) after having experienced a claustrophobic event directly before answering the questionnaire. In comparison, only 63.8% of patients without a claustrophobic event suggested the upright open scanner (32.8%; 38/116) or the panoramic open scanner (31%; 36/116) as their first choice for future development. Patients without an immediate claustrophobic event chose a short-bore (19.8%; 23/116) and open one-column design (16.4%; 19/116) more often than patients with a claustrophobic event in the preceding MRI examination (Table 3; Fig. 4). The difference in preferences of patients with a claustrophobic event in favor of the upright open and open panoramic scanner designs over the short-bore and open one-column scanners compared with patients without a claustrophobic event was statistically significant (*p* = 0.047; Table 3; Fig. 4).

**Table 3:** Preferred scanner design for future scanner development depending on the presence of a claustrophobic event

Preferred scanner design for future development	In patients without a claustrophobic event after MRI		In patients with a claustrophobic event after MRI		Change in preferences depending on the presence of a claustrophobic event
					*p* = 0.047 ***
	*n* = 116	95% CI	*n* = 44	95% CI	
A: Open panoramic scanner, *n* (%)	36 (31.0)	23.3–39.9	17 (38.7)	25.7–53.4	
B: Short-bore scanner, *n* (%)	23 (19.8)	13.6–28	3 (6.8)	2.3–18.2	
C: Open one-column scanner, *n* (%)	19 (16.4)	10.7–24.2	3 (6.8)	2.3–18.2	
D: Upright open scanner, *n* (%)	38 (32.8)	24.9–41.7	21 (47.7)	33.8–62.1	

Patients who experienced a claustrophobic event directly before completing the questionnaire preferred an upright open scanner and open panoramic design significantly more often than a short-bore design. The change in difference depending on the presence of a claustrophobic event was statistically significant (*p* = 0.047)

***p value was calculated with Pearson’s chi-square test

### Influence on design preferences of the scanner type patients were scanned in

**Fig. 5:**
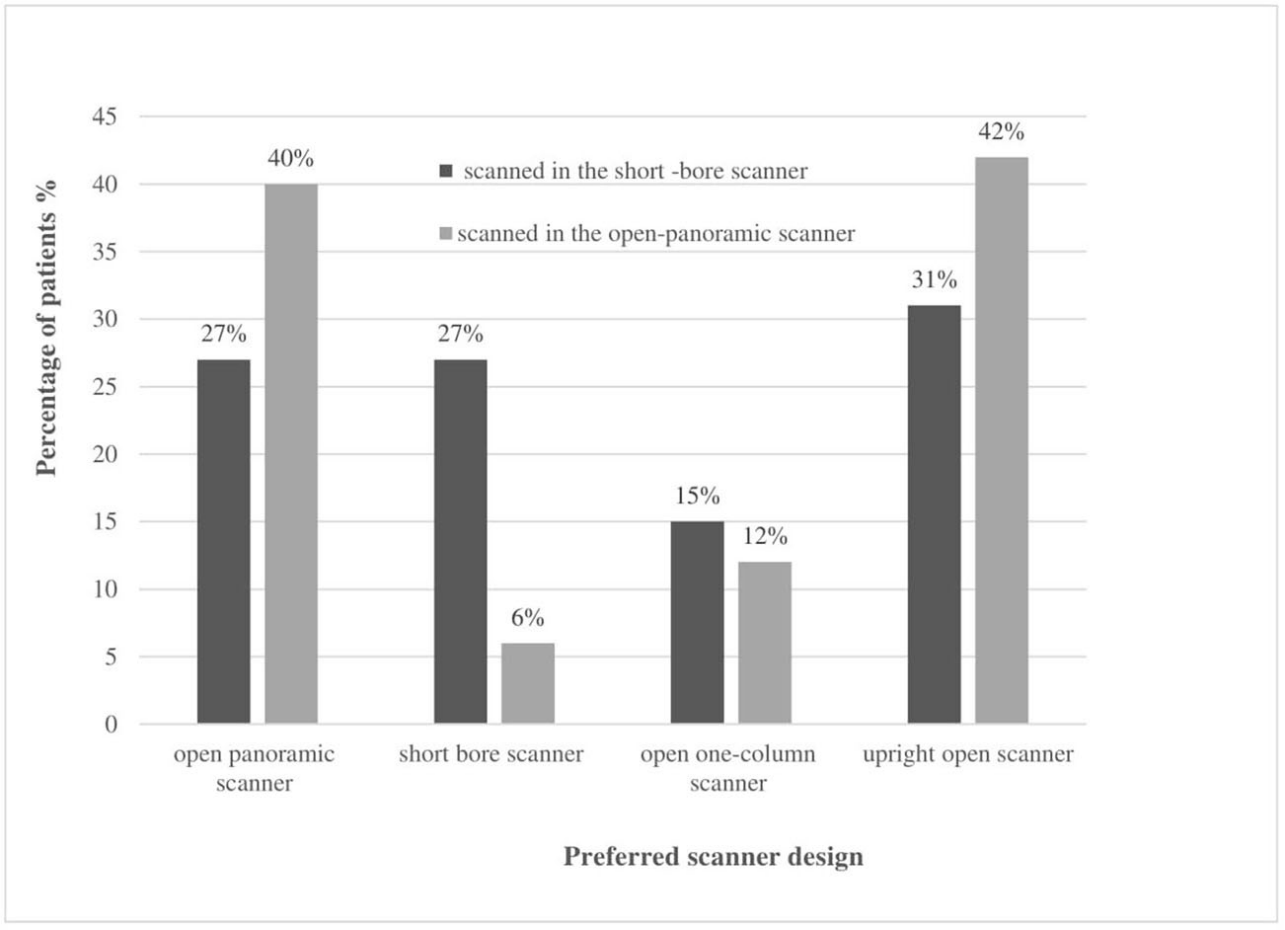
Design preferences in relation to the scanner type patients were scanned in directly before filling out the questionnaire. Patients scanned in a short-bore scanner within the study protocol preferred the short-bore scanner design for future development significantly more often than patients scanned in the open panoramic scanner (27% vs. 6%). The changes in design preferences depending on the scanner type patients were scanned in were significant (*p* = 0.003)

Patients most often preferred the upright open scanner for future development whether they were scanned in the short-bore scanner (25/79, 31%) or open panoramic scanner (34/81, 42%). Patients scanned in the short-bore scanner ranked the short-bore scanner and open panoramic scanner equally in the second place (21/79, 27% each). In contrast, patients scanned in the open panoramic scanner preferred the open panoramic scanner with 40% (32/81) over the short-bore scanner (5/81, 6%). The difference in preferences depending on the scanner type patients were scanned in was significant (for all *p* = 0.003; Fig. 5).

### Suggested improvements for future scanner design

**Fig. 6:**
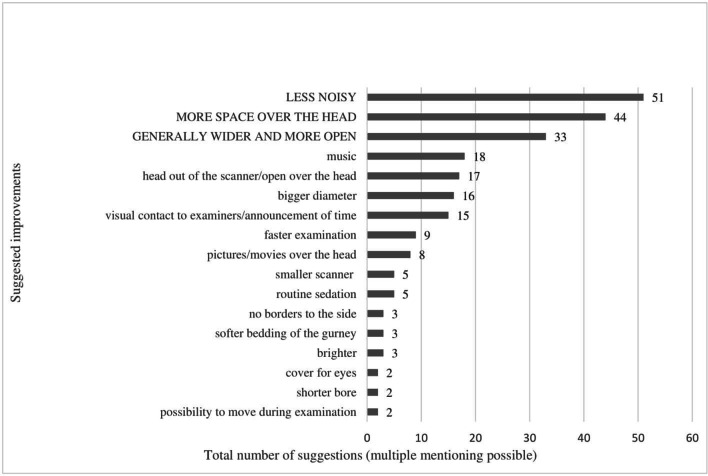
Suggested improvements by patients at higher risk for claustrophobia for future scanner development. Most patients suggested a reduction of noise (31.9%, 51/160), more space over their head (27.5%, 44/160), and overall more space (20.6%, 33/160). Patients could make multiple suggestions. Not shown are the following other suggestions that were mentioned only once: cooler, adjustable air conditioning, no breaks in the examination, broader gurney, sitting scanner position, open casing of the scanner, active warming of hands and feet, and opportunity to leave the scanner anytime

When asked which specific improvements they would prefer for future scanner development, most patients suggested a reduction in noise (31.9%, 51/160), more space over their head (27.5%, 44/160), and overall more space (20.6%) (Fig. 6).

## Discussion

We analyzed scanner design preferences in patients at high risk for claustrophobia. To our knowledge, we were the first to selectively study MRI scanner design preferences based on the first visual perception in this study population.

Our aim was to gain insight into possible ways to reduce claustrophobia in future MRI examinations for claustrophobic patients. To this end, we focused on the role of visual scanner design features and other factors influencing cognitive attitudes towards an MRI scanner such as information about its diagnostic performance or previous MRI experiences. The insights gained can be useful for informed referral of claustrophobic patients to a specific MRI scanner as well as for further industrial MRI scanner developments.

A majority of patients preferred more open panoramic and upright open scanners for further development as opposed to further development of short-bore designs. Preferences for a short-bore design in an upcoming examination could be significantly increased after education about the better diagnostic performance of this otherwise visually less-attractive design. Preference for this scanner design was also higher among patients who completed an examination in a short-bore scanner without a claustrophobic event.

The general preference of more open as opposed to short-bore designs we found in our patients is in line with previous studies reporting a reduction in claustrophobia with open scanners [22]. Bangard et al found less anxiety and better acceptance of MRI examinations in an open panoramic scanner in patients with a history of a claustrophobic event as in a closed-bore scanner [21]. However, the use of closed designs with a shorter bore was also reported to significantly reduce the claustrophobic event rate compared with older closed-, long-bore scanners [5, 23].

However, in another study, neither an open panoramic nor a short-bore scanner design was found to be superior in reducing the claustrophobic event rate in a population of patients at high risk for claustrophobia with a disappointingly high rate of claustrophobic events of more than 25% [19].

Since the conclusion of our study in 2009, there were many new developments such as scanners with even shorter and wider closed bores, the advent of pulse sequences that shorten image acquisition, and further noise reduction. However, those latest state-of-the-art MRI scanners are not available everywhere, partially due to their comparatively higher costs.

Individual claustrophobic patients might benefit, though, from a referral to a scanner with features that might help to reduce claustrophobia-related distress as much as possible.

As our study shows, claustrophobic patients visually prefer open- over closed-bore designs and mention noise as a major subjective concern. This can be explained by previously reported findings suggesting that negative cognitions before an MRI examination and even just looking at the scanner can trigger claustrophobic events and anxiety [17–19]. Evidence of an increase in cortisol levels as part of an anticipatory stress reaction prior to MRI examinations [24] further underlines the importance of the visual perception of a scanner.

Therefore, we conclude that the choice of a scanner design for an individual claustrophobic patient, whenever possible, should focus on the provision of the two design items patients most often mentioned, i.e., “open bore” and “less noise,” to reduce claustrophobic experiences.

If a referral to such a scanner is not possible, e.g., for logistic reasons, our results indicate that educating a patient about a better diagnostic performance of an otherwise subjectively less-attractive scanner design (e.g., a closed-bore design) might help to make the scanner more acceptable to the patient. In line with this, a reduction of claustrophobia by patient education about the scheduled MRI examination has been established before [7, 25]. Further, Hyde et al report that even patients not at high risk for claustrophobia complain of too little patient-centered information before MRI examinations [26].

We also found that patients scanned in the short-bore scanner without a claustrophobic event accepted this particular scanner design visually more readily than patients scanned in an open panoramic scanner. This finding could be attributable to decreased phobic avoidance after situational claustrophobic exposure [27] and reduced restriction subscale scores in the CLQ in MRI examinations without a claustrophobic event [28]. Practically, this could mean that patients with a history of a successful MRI scan in a short-bore scanner have a reasonable chance to successfully complete a further examination in this scanner type again. However, we have not considered previous scanning experiences of patients before our study, which could result in a bias in our results.

Nevertheless, we still think that a thorough history of acute anxiety levels and previous MRI experiences could help to choose an appropriate scanner design for an individual patient at high risk for claustrophobia.

We acknowledge that our study also has limitations.

Firstly, since the conduct of our study, there have been a host of new developments with regard to scanner designs, scanner features such as noise reduction and shorter acquisition times, and new approaches to relieve claustrophobia such as virtual reality tools. Secondly, our study relies on preferences the patients voiced after seeing sketches of scanner designs as opposed to the actual experience of lying within the scanner or simulation of the experience using newly available virtual reality simulation tools.

However, we still think that our data are valuable, as the newly developed scanners that have become available since the conduct of our study are quite expensive and not available everywhere worldwide. Hence, if a physician wants to refer a claustrophobic patient for an MRI examination in a newer scanner to relieve claustrophobia, our data can be helpful in choosing the most promising option if newer scanners are not available in an acceptable distance.

Moreover, as our drawings of the scanners are quite minimalistic, the sketch of the closed-bore scanner in our study could also mimic the first glimpse look of newer closed-, slightly wider, and shorter bore imagers.

Even though we used 2-dimensional sketches of designs, we think that, given the well-established influence of cognitions and the first glimpse of a scanner design on the occurrence of claustrophobia, our results are still useful and can guide future development by manufacturers. This is especially true because our study shows that claustrophobic patients clearly prefer open designs while recent hardware developments have focused to a huge part on shortening and widening closed-bore scanners.

As an advantage of our study, to our knowledge, this is the first study that selectively included patients at high risk for claustrophobia and can therefore add valuable insight into claustrophobia in MRI. New techniques, such as virtual reality simulation, would be an excellent tool to follow-up on our data and confirm preferences of claustrophobic patients.

## Conclusion

Patients at high risk for claustrophobia prefer open panoramic and seated upright open scanner designs for future development by the industry.

Education about a better diagnostic performance of a given scanner can help improve acceptance of otherwise visually less-attractive scanner designs for an upcoming examination in claustrophobic patients. Less noise and more space within the scanner were the most commonly stated wishes of patients regarding the reduction of claustrophobia. Claustrophobic patients with a history of a successful MRI scan in a short-bore scanner are significantly more likely to accept this design. Overall, our findings can guide the referral of claustrophobic patients to existing scanners as well as future industrial scanner development.

## Electronic supplementary material

ESM 1 (PNG 3847 kb)

High resolution image (TIFF 204 kb)

ESM 2 (PNG 4049 kb)

High resolution image (TIFF 214 kb)

ESM 3 (PNG 1941 kb)

High resolution image (TIFF 149 kb)

ESM 4 (TIFF 16.2 kb)

## Abbreviations

CLQ: Claustrophobia Questionnaire
MRI: Magnetic resonance imaging
