# Supplementary figures and images for: Patient preferences for development in MRI scanner design: a survey of claustrophobic patients in a randomized study
Source: Eur Radiol. 2020 Sep 2;31(3):1325–35. doi: 10.1007/s00330-020-07060-9 (PMC7880963; doi:10.1007/s00330-020-07060-9)

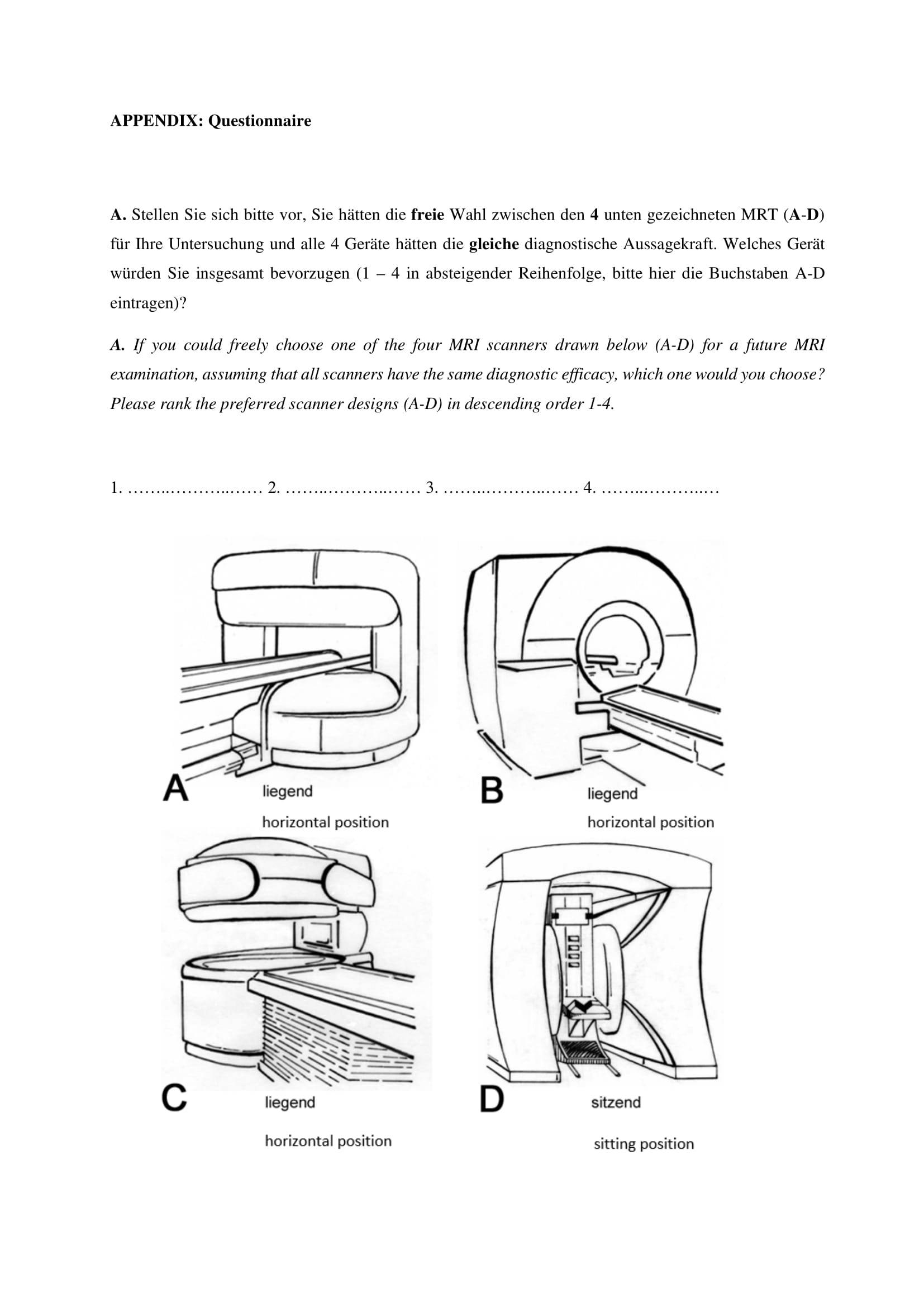

Supplement: Supplementary file 2 — High resolution image (TIFF 204 kb) [file 330_2020_7060_MOESM1_ESM.tiff]

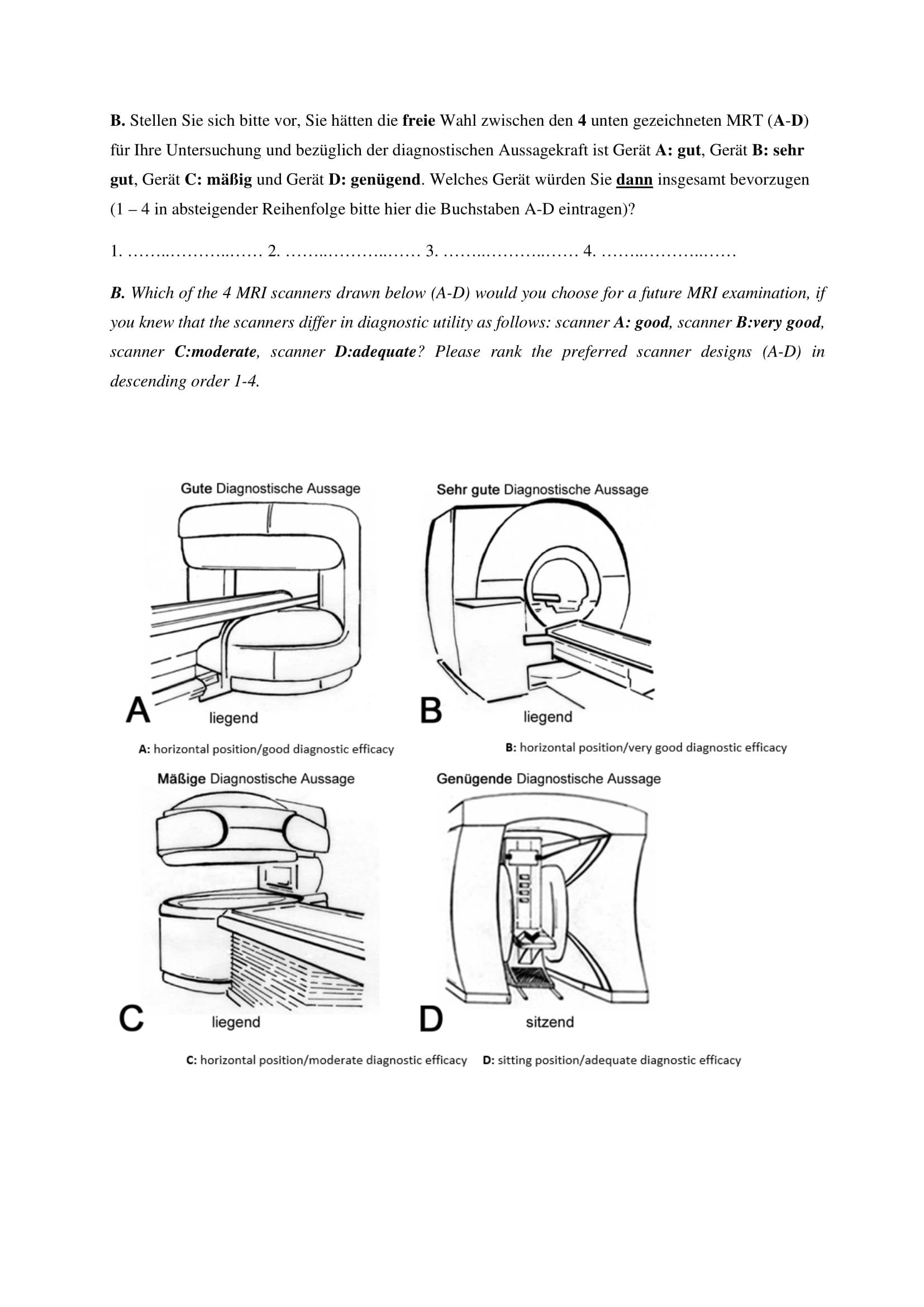

Supplement: Supplementary file 4 — High resolution image (TIFF 214 kb) [file 330_2020_7060_MOESM2_ESM.tiff]

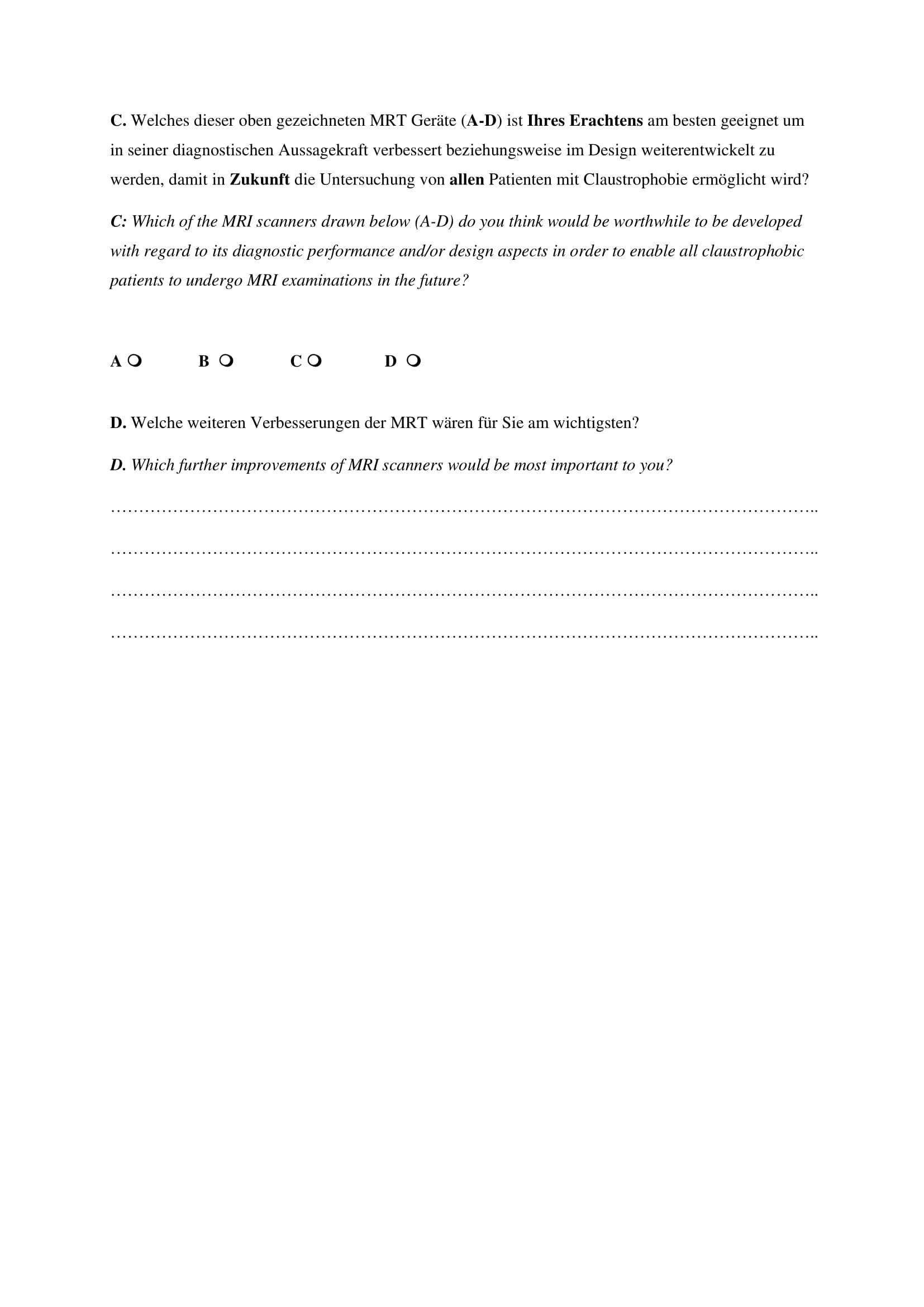

Supplement: Supplementary file 6 — High resolution image (TIFF 149 kb) [file 330_2020_7060_MOESM3_ESM.tiff]
