# Supplementary material for: Patient preferences for development in MRI scanner design: a survey of claustrophobic patients in a randomized study
Source: Eur Radiol. 2020 Sep 2;31(3):1325–35. doi: 10.1007/s00330-020-07060-9 (PMC7880963; doi:10.1007/s00330-020-07060-9)
Supplement: Supplementary file 7 — ESM 4 (TIFF 16.2 kb) [file 330_2020_7060_MOESM10_ESM.docx]

***TABLE 4:***

**Preferred scanner design for future development depending on the scanner they were scanned in (short-bore or open panoramic) before answering the question**

| **Preferred scanner design for future development** | **answers to question 3 in patients scanned in the short -bore scanner** | | **answers to question 3 in patients scanned in the open-panoramic scanner** | | **change in preferences depending on the scanner they were scanned in** |
| --- | --- | --- | --- | --- | --- |
|  |  |  |  |  | **p = 0.003***** |
|  | **n=79** | **95%CI** | **n=81** | **95%CI** |  |
| **A: open panoramic scanner n (%)** | 21(27.0) | 18.1-37.2 | 32(40.0) | 29.6-50.4 |  |
| **B: short bore scanner n (%)** | 21(27.0) | 18.1-37.2 | 5(6.0) | 2.7-13.6 |  |
| **C: open one-column scanner n (%)** | 12(15.0) | 8.9-24.7 | 10(12.0) | 6.8-21.3 |  |
| **D: upright open scanner n (%)** | 25(31.0) | 22.4-42.5 | 34(42.0) | 31.8-52.8 |  |

Patients scanned in the short-bore scanner preferred the short-bore design for future development significantly more often than patients scanned in an open panoramic scanner (p= 0.003).

*** p-values were calculated with Pearson´s Chi square test
